# Could Repeated Cardio-Renal Injury Trigger Late Cardiovascular Sequelae in Extreme Endurance Athletes?

**DOI:** 10.1007/s40279-022-01734-8

**Published:** 2022-07-18

**Authors:** Johannes Burtscher, Paul-Emmanuel Vanderriele, Matthieu Legrand, Hans-Georg Predel, Josef Niebauer, James H. O’Keefe, Grégoire P. Millet, Martin Burtscher

**Affiliations:** 1 Department of Biomedical Sciences, University of Lausanne, Lausanne, Switzerland; 2 Institute of Sport Sciences, University of Lausanne, Lausanne, Switzerland; 3 Department of Anesthesia and Perioperative Care, Division of Critical Care Medicine, University of California, San Francisco, CA USA; 4 Department of Preventive and Rehabilitative Sport Medicine and Exercise Physiology, Institute for Cardiology and Sports Medicine, German Sport University, Cologne, Germany; 5 University Institute of Sports Medicine, Prevention and Rehabilitation, Paracelsus Medical University Salzburg, Salzburg, Austria; 6 Saint Luke’s Mid America Heart Institute, University of Missouri-Kansas City, Kansas City, MO USA; 7 Medical Section, Department of Sport Science, University of Innsbruck, Fürstenweg 185, 6020 Innsbruck, Austria

## Abstract

Regular exercise confers multifaceted and well-established health benefits. Yet, transient and asymptomatic increases in markers of cardio-renal injury are commonly observed in ultra-endurance athletes during and after competition. This has raised concerns that chronic recurring insults could cause long-term cardiac and/or renal damage. Indeed, extreme endurance exercise (EEE) over decades has sometimes been linked with untoward cardiac effects, but a causal relation with acute injury markers has not yet been established. Here, we summarize the current knowledge on markers of cardiac and/or renal injury in EEE athletes, outline the possible interplay between cardiac and kidney damage, and explore the roles of various factors in the development of potential exercise-related cardiac damage, including underlying diseases, medication, sex, training, competition, regeneration, mitochondrial dysfunction, oxidative stress, and inflammation. In conclusion, despite the undisputed health benefits of regular exercise, we speculate, based on the intimate link between heart and kidney diseases, that in rare cases excessive endurance sport may induce adverse cardio-renal interactions that under specific, hitherto undefined conditions could result in persistent cardiac damage. We highlight future research priorities and provide decision support for athletes and clinical consultants who are seeking safe strategies for participation in EEE training and competition.

## Key Points

**Table:** 

Clear evidence for long-term cardiac and/or renal damage resulting from chronic recurring insults from extreme endurance exercising is still missing.
A better understanding of this association is important to enable endurance athletes to reap all the health benefits from exercising without risk of cardiac/renal adversities.

## Introduction

*Case*

GF is a 58-year-old male with intermittent atrial fibrillation (AF), medically treated mild hypertension, and stage 3 chronic kidney disease (CKD), with a creatinine of 132.6 μmol/L and an estimated glomerular filtration rate (eGFR) of 48 mL/min/1.73 m^2^. For the past 20 years his creatinine has typically ranged from 115 to 132.6 μmol/L. Currently, he has no signs or symptoms of renal or cardiac disease despite his stage 3 CKD.

He has been an avid exerciser most of his adult life and began training for and competing in endurance exercise events at age 35 years, at which time his renal function was normal. Due to an orthopedic injury, he is currently doing only light exercise in the form of walking and recreational bicycling totaling not more than 3 h/week. Renal workup was largely unremarkable showing no vasculitis, glomerulonephritis, renal cysts, kidney stones, or renal artery stenosis, and the patient did not have a family history of kidney disease.

In the past he had typically performed several marathons and half marathons each year, occasionally competed in ultramarathon events, and over the last decade he has completed seven full-distance Ironman^®^ triathlons. On two occasions following extreme exercise efforts (a 50 km ultramarathon and an Ironman^®^ triathlon) he developed rhabdomyolysis with acute kidney injury requiring hospitalization.

Could repeated participation in extreme endurance competitions have led to cumulative kidney injury, and could this increase the risk of permanent cardiac disease for this patient?

Cases like the one described above raise the question whether extreme endurance exercise (EEE) under certain conditions may increase risks for cardiovascular (CV) and/or renal damage. In light of the impressive general benefits of exercise, it is of utmost importance to identify these conditions and dispel concerns in the absence of relevant risk factors.

Regular physical activity (PA), including exercise, which is defined as planned, structured, repeated, and goal-directed PA, is among the most important lifestyle factors for positively influencing cardiorespiratory fitness, healthy aging, and longevity [1, 2]. Public health guidelines recommend at least 150–300 min of PA at moderate aerobic intensity or 75–150 min at vigorous intensity per week [2]. However, much more PA may be necessary to achieve maximal health benefits. Indeed, a large prospective cohort study demonstrated that maximal longevity gains can be found at about 700 min of moderate or 350 min of vigorous activity per week [3]. The increased life expectancy of elite athletes and especially endurance athletes [4], as compared with the general population, indicates that elite sports—thus involving exercise at high intensity and volume and in combination with competition-related stressors—might also reduce all-cause mortality.

Despite the pronounced CV as well as general health benefits conferred by regular PA, an increasing number of reports raise concerns about detrimental health effects associated with long-term EEE, i.e., life-long marathon (26.2 miles) and/or ultramarathon (any running event > 26.2 miles or 42.2 km) running [5–7]. For example, post-mortem analyses in highly trained athletes revealed left ventricle (LV) hypertrophy and interstitial myocardial fibrosis [5]. These authors suspected that life-long, repetitive bouts of demanding PA resulted in fibrous replacement of the myocardium, likely generating the pathological substrate for the propagation of fatal arrhythmias [5]. Others suggested that the extraordinary hemodynamic challenge (disproportionate increase in afterload and wall stress) of the right heart chambers may result in myocardial fatigue or damage when intense exercise is sustained for prolonged periods [6].

Generally, the heart adapts physiologically and anatomically in response to chronic endurance exercise (athlete’s heart), but in occasional cases, maladaptive processes may result in pathologic remodeling, myocardial fibrosis, and/or arrhythmias [5, 6, 8]. Transient elevations in blood markers of cardiac (e.g., cardiac troponin) and/or renal injury (e.g., serum creatinine) are commonly observed after long-distance running [9, 10]. However, long-term consequences of repeated acute events and the role of potential cardio-renal feedback loops remain unknown, primarily due to the inherent complexity of appropriate study designs. Even so, both the predictive importance of creatinine levels for troponin elevations after marathon running [11] and the well-known association between heart and kidney damage (cardio-renal syndrome) [12] suggest the existence of such a relationship [13].

The investigation of possible causal associations between clinical observations and long-term high-intensity and/or high-volume endurance exercise is an important future endeavor to allow athletes to safely practice health-promoting exercise. This review provides a summary of common exercise-induced increases in markers of cardio-renal injury, and evaluates the hitherto poorly understood link between permanent heart damage and kidney injury, in an attempt to lay the groundwork for future studies as well as to provide guidance for athletes and clinical consultants on which parameters to consider when advising individuals who are engaging in EEE.

## Myocardial and Renal Injuries Following Extreme Endurance Exercise (EEE)

### Potential Myocardial Injury Due to Marathon and Ultramarathon Running

Markers indicating potential myocardial injury (elevated serum creatine kinase MB isoenzyme levels; CK-MB) have already been reported in early studies (during the 1980s) that evaluated marathon runners post competition [14–16]. Mean serum CK-MB of 35 runners, measured 24 h after finishing the race, was 130 IU/L (normal values are below 5 IU/L) or 8.3% of total CK activity [14]. Such high values would usually indicate substantial myocardial necrosis, but myocardial scintigraphy with technetium Tc 99 m pyrophosphate, which was performed in 12 marathon runners, did not reveal any myocardial injury. These authors demonstrated in another study (based on skeletal muscle biopsies) that the elevated serum CK-MB activity very likely arises from a non-cardiac source, i.e., from skeletal muscle [15]. An increased CK-MB was subsequently found in up to 95% of marathon finishers [16].

With the advent of cardiac troponin I (cTnI) and T (cTnT) assays, the diagnosis of myocardial damage has become more accurate [17]. However, first-generation cardiac troponin assays still displayed elevated values in a certain proportion of marathon runners without clinically significant cardiac damage [18]. In a meta-analysis published in 2007, significant exercise-induced cTnT release was measured in almost half of the endurance athletes studied, primarily in marathon runners [19]. These findings were confirmed by another subsequent meta-analysis, in which cTnI levels were demonstrated to be less commonly elevated than cTnT levels [10]. The clinical relevance of these cTn elevations after marathon running remained unclear and some results are controversial, likely also due to the use of first- or second-generation cTn assays and the use of different cutoffs. A study comparing effects of marathon running on second-generation and third-generation cTn assays also included measurements of N-terminal pro-brain natriuretic peptide (NT-proBNP) for additional information on myocardial wall stress [20]. The authors reported increases in NT-proBNP that were independent of the elevations of cTnT and cTnI.

The time course of elevated cTn levels after prolonged exercise (peak values between 2 and 6 h after exercise) is different to that caused by myocardial infarction (peak values between 10 and 12 h after an ST segment-elevation acute myocardial infarction) [21]. The early releasable cTn pool is considered the primary origin of release. Conversely, increased cTn levels caused by irreversible myocyte injury with apoptosis and/or necrosis stem from cTn complexes, which are structurally bound to the actin filament and represent the majority of cTn [21, 22].

**Fig. 1:**
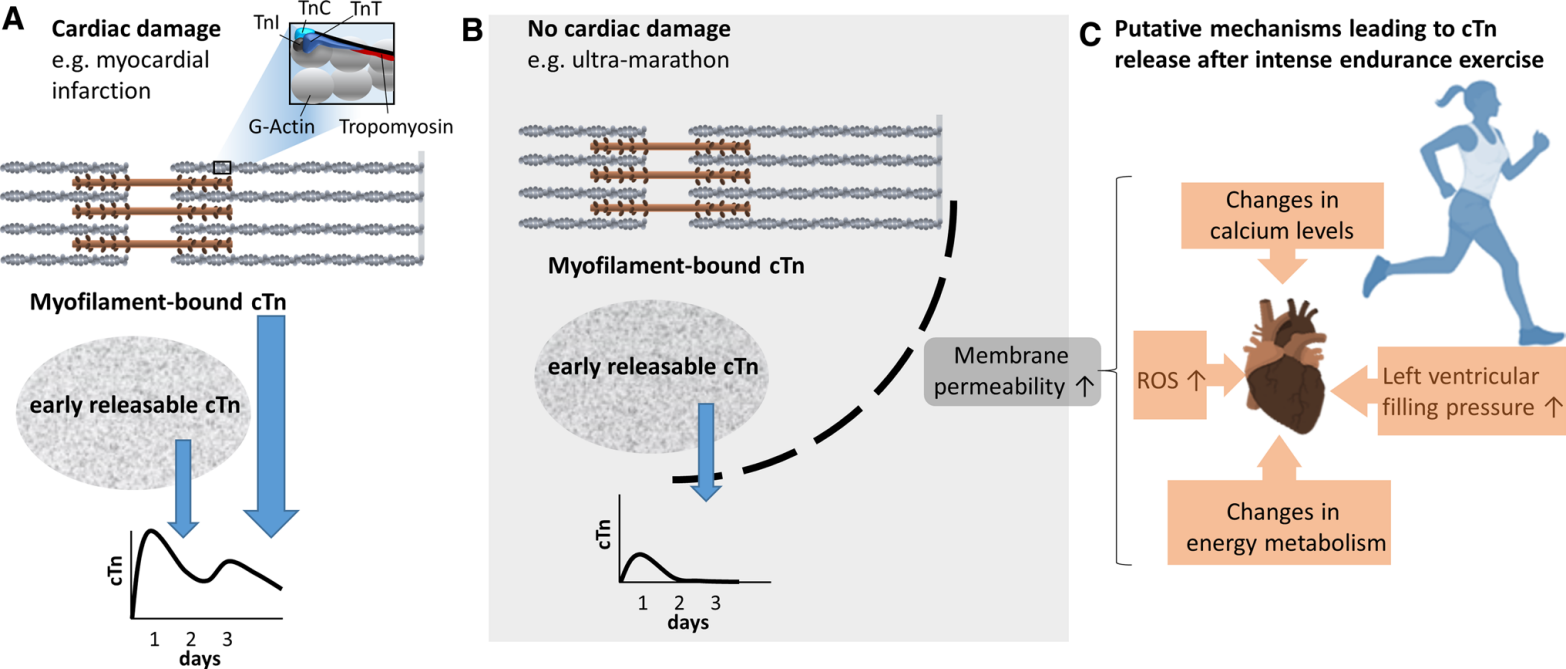
Potential triggers for “myocardial injury” in endurance athletes [22, 23, 25]. While a substantial amount of released cardiac troponin (cTn) from myofilaments indicates cardiac damage (**A**), the early releasable cTn can occur in absence of persistent cardiac damage (**B**). Metabolic stress associated with intensive endurance exercise may facilitate cardiomyocyte membrane permeability by increased metabolic energy demand, reactive oxygen species (ROS) production, perturbed calcium homeostasis, and increased filling pressures (**C**). The resulting cTn release is usually modest and of short duration

The biochemistry, prevalence, and potential mechanisms of exercise-induced cardiac troponin elevation have been extensively reviewed (Fig. 1) [21, 23]. Likely mediated by mechanisms such as oxidative stress and/or alterations in pH, calcium, and energy metabolism, intensive aerobic exercise will cause a transient increase in membrane permeability of cardiomyocytes [24] and the resulting release of proteins, including cTn from the early releasable pool. Recently, in animal studies (swine), preload-induced mechanical stress of the LV was shown to cause myocyte injury with apoptosis that may lead to release of proteolytic degradation products of cTn [25]. Regardless of its cause, an elevated cTn remains a matter of concern as it is associated with a relatively worse prognosis not only in cardiac patients but even in asymptomatic cross-sectional populations [26]. Thus, potential clinically relevant consequences of repeated increases in markers of myocardial injury associated with EEE, especially if accompanied by additional pathophysiological processes, require further elucidation.

Galectin-3, a potential marker of cardiac fibrosis, is another protein upregulated by aerobic exercise [27], including by running a 60 km ultramarathon [28]. While an increase of galectin-3 may indicate alterations in cardiac structure, in endurance athletes it was not correlated with cardiac function or myocardial fibrosis [27]. Similar to CK-MB, the galectin-3 rise following exercise may also have its origin in the skeletal musculature. Galectin-3 is thought to be a specific marker of pathological processes like inflammation or fibrosis rather than an organ-specific marker [29]. Myocardial fibrosis (late gadolinium enhancement) was found in up to 50% of endurance athletes, more frequently in those with higher cumulative endurance training experience and completed marathons [30], and was potentially associated with an increased risk of cardiac arrhythmias and mortality [31]. Fibrosis was typically observed at the insertion of the right ventricle into the septum, indicating mechanical stress rather than ischemia as a causative factor [6], and thus rendering an association with sudden cardiac death less likely. Suppression of tumorigenicity 2 (ST2) is another marker for cardiac fibrosis. In contrast to galectin-3, ST2 remained elevated for even 3 h after competition in marathon or ultramarathon (67 km) races [7]. The elevation of these cardiac biomarkers is usually not associated with either systolic or diastolic dysfunction and has been suggested to be physiological [32].

Notably, LV systolic function in swine was preserved after repetitive pressure overload despite significant myocyte apoptosis, but was associated with myocardial remodeling characterized by myocyte hypertrophy and interstitial fibrosis [33]. In particular, right ventricular (RV) dysfunction due to repetitive volume overload and incomplete recovery with chronic EEE was found to represent a substrate for proarrhythmic RV remodeling in some highly trained athletes [34]. Taken together, elevated cardiac biomarkers usually are reversible non-pathological events, but may become detrimental in some cases of chronic EEE, as evidenced by the predictive importance of exercise-induced elevation of cTn for CV events in older long-distance walkers [21].

### Potential Kidney Injury due to Marathon and Ultramarathon Running

**Fig. 2:**
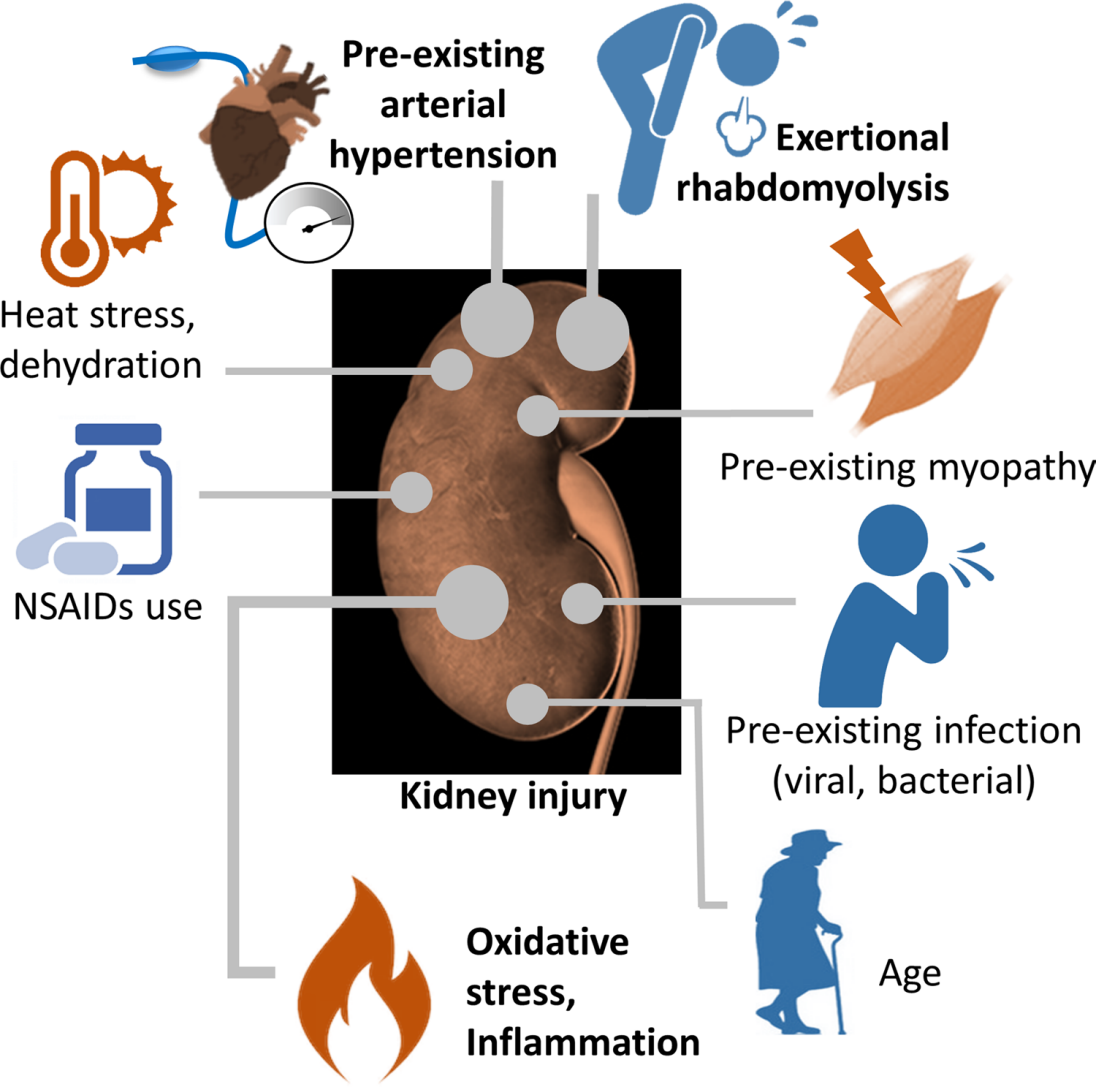
Potential triggers of kidney injury in extreme endurance athletes. *NSAIDs* nonsteroidal anti-inflammatory drugs

Urinary abnormalities were observed in 20 participants of the 1941 Boston marathon and indicated potential kidney injuries in finishers. The authors of that study reported large numbers of casts in most of the urine samples and albumin in various quantities [35]. Subsequently, rare cases (e.g., ten cases over 9 years from the Comrades Marathon; three cases required hemodialysis and one had peritoneal dialysis [36]) meeting the diagnostic criteria for acute renal failure have been reported in long distance runners, secondary to rhabdomyolysis (i.e., a breakdown of muscle tissue and consequential release of damaged muscle protein into the blood) associated with hemoglobinuria and myoglobinuria [36, 37], occasionally even requiring hemodialysis [36–38]. Rhabdomyolysis, dehydration, heat stress, hypotension, nonsteroidal anti-inflammatory drugs (NSAIDs), and hyperuricemia [39], in addition to pre-existing diseases/infections [38] may all contribute to the multifactorial pathophysiology of acute kidney injury (AKI) in extreme endurance athletes (Fig. 2).

Beside more severe adverse events, about 40% of marathon [40, 41] and 30–85% of ultramarathon [42, 43] participants may experience a transient or prolonged rise in serum creatinine and/or reduced eGFR meeting the criteria of AKI [44]. Although this observation caused concern based on the well-established association of AKI and subsequent morbidity and mortality [12], its clinical relevance in EEE still remains a matter of debate [45, 46].

While AKI after prolonged running was previously considered as the primary consequence of skeletal muscle destruction, dehydration, and heat stress [38], more recent findings point to additional intrinsic and independent factors contributing to the renal damage [9]. In a cohort of 38 runners whose blood had been analyzed after multiple endurance races, runners who met AKI criteria during the first race (n = 16) had a higher risk of meeting AKI criteria again during subsequent races [45]. For nine of these 16 runners, serum creatinine elevations and impairments of eGFR were attenuated in later races. No evidence for cumulative kidney injury across different races was found in this study [45]. Recently, a 2.7-fold increase (from pre- to post-race, 120 km ultramarathon) of urinary neutrophil gelatinase-associated lipocalin was observed, a novel biomarker for renal disease and particularly important in the early detection of an AKI [46]. However, in this observational study of adequately hydrated athletes who did not consume NSAIDs, no evidence for clinically important kidney damage was found based on the RIFLE (Risk, Injury and Failure; and Loss, and End-stage kidney disease) criteria [46]. Nevertheless, 11 case report publications (27 individuals) of severe AKI, commonly associated with systemic illness or nephrotoxic medications [42], still need attention. In summary, there is no evidence for a cumulative effect of repeated mild-to-moderate AKI following (ultra)marathon running, but rare cases of more severe AKI have been reported [42] and related long-lasting effects due to a combination of several factors cannot be excluded based on evidence of long-term impact of AKI in other settings.

### Association Between Heart and Kidney Damage

**Fig. 3:**
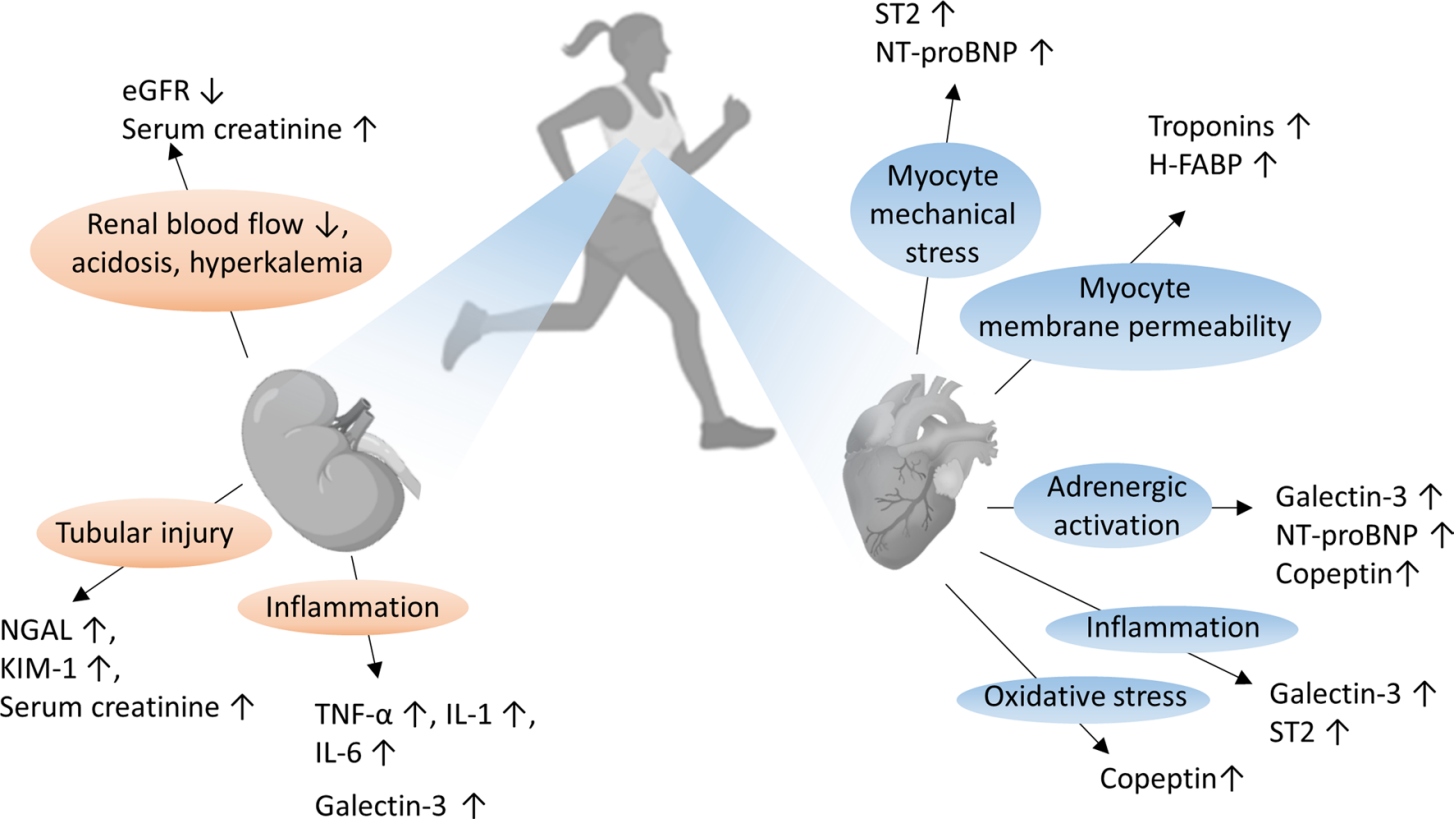
Summary of renal and cardiac injury markers as a consequence of extreme endurance exercise (EEE; see text and [12]). *eGFR* estimated glomerular filtration rate, *NGAL* neutrophil gelatinase-associated lipocalin, *KIM-1* kidney injury molecule 1, *TNF-α* tumor necrosis factor α, *IL-1 and -6* interleukins 1 and 6, *ST2* suppression of tumorigenicity 2, *NT-proBNP* N-terminal pro-brain natriuretic peptide, *H*-*FABP* heart-type fatty acid binding protein

Generally, the association between heart and kidney damage is well established [12]. An elevated systemic blood pressure, observed in 7–8% of ultramarathon runners (4–5% were not taking antihypertensive medication) [47] may negatively impact on both the heart and the kidney [48]. Systemic hypertension is a well-established risk factor for the development of heart diseases, including the onset and progression of AF [49], and it may also cause renal damage [50], initiating the vicious cycle of cardio-renal syndrome [12]. Damage to one of these organs is often associated with dysfunction or damage of the other, due to the cardio-renal crosstalk [12]. An increase of a multitude of biomarkers in response to (extreme) endurance exercise (Fig. 3), which potentially could lead to adverse cardio-renal consequences in the long-term, has been reported.

**Fig. 4:**
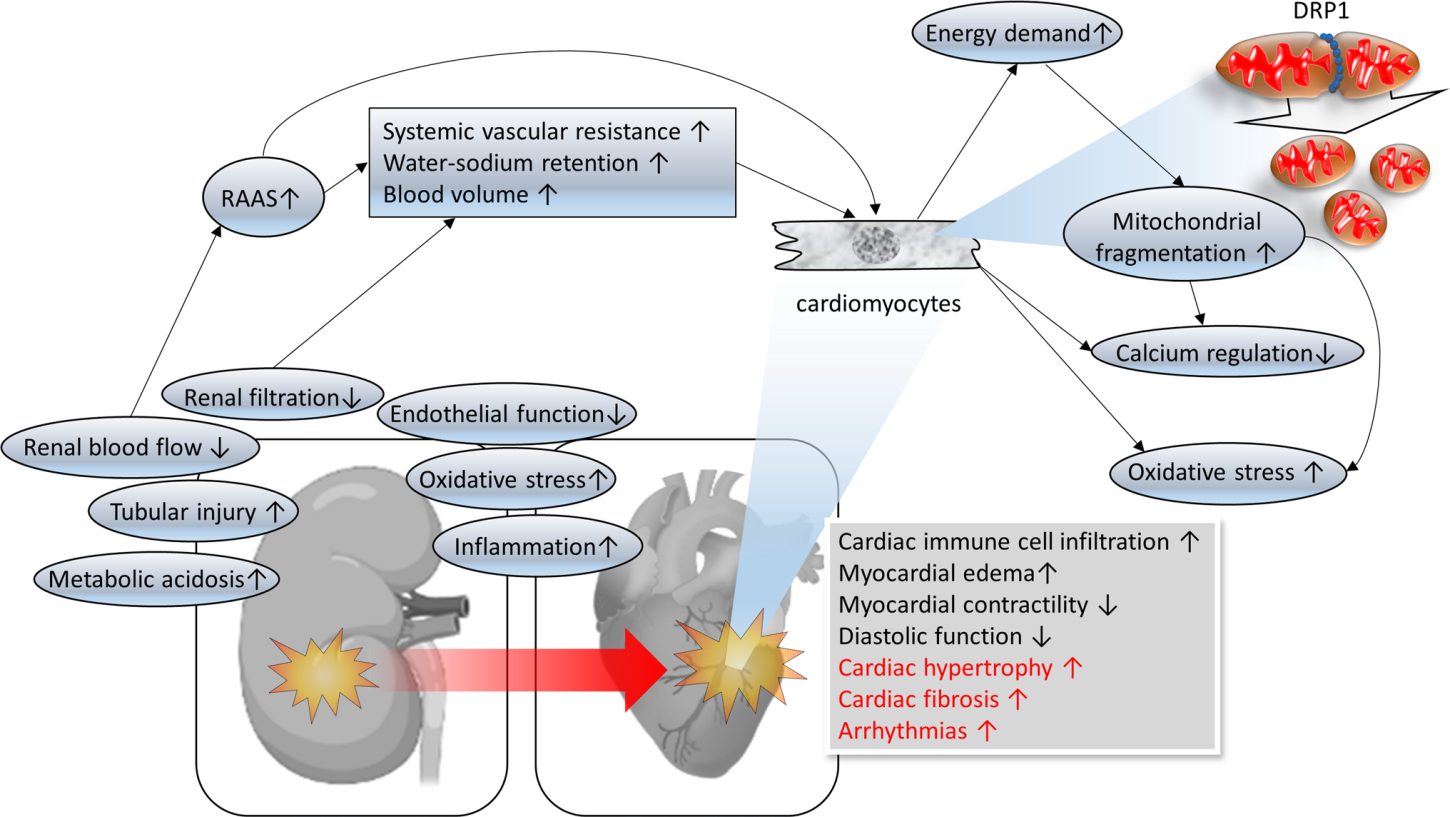
Putative mechanisms of cardiac damage following renal dysfunction. *RAAS* renin–angiotensin–aldosterone system, *DRP1* dynamin-related protein 1

Although not related to athletes, a large meta-analysis (including 25 studies involving 254,408 adults; 55,150 with AKI) revealed a markedly increased risk of death from CV causes among patients suffering from AKI [51]. In this study, AKI was associated with an 86% and a 38% increased risk of CV mortality and major CV events, respectively, confirming the link between cardiac and renal disease [51]. Also the risk of CV events and in particular of AF increases with lower eGFR [52]. A graded association was demonstrated even within the 90–130 ml/min/1.73 m^2^ range [52]. The prevalence of AF gradually increases with impaired eGFR [53], but whether and how the mostly transient eGFR impairment post EEE [44, 54] and the higher prevalence of AF in marathon runners (5 vs. 0.7 in controls) [55] are associated remains to be elucidated [56]. The putative role of cellular mechanisms for cardiac injury following AKI are summarized in Fig. 4.

Although not fully understood, the cardiac consequences of AKI seem to be mediated predominantly by systemic inflammation, cardiac immune cell infiltration, activation of the renin–angiotensin–aldosterone system (RAAS), neuro-hormonal activation, oxidative stress, dysregulated calcium homeostasis, and endothelial dysfunction following AKI, all of which may induce mitochondrial dysfunction, cardiac hypertrophy, and fibrosis in the heart [12, 57–60]. AKI-induced damage to the heart is associated with RAAS and sympathetic nervous system activation [61]. The latter increases the energy demand of cardiomyocytes and can perturb the cellular homeostasis, for example, calcium fluxes [62]. RAAS activation indirectly (e.g., due to fluid balance and blood volume regulation) and directly impairs cardiac function following renal injury [62].

An increase of systemic inflammation has been observed in animal models of ischemic AKI, as recently reviewed [12]. AKI in rodent models furthermore induces cardiac mitochondrial injury that is mediated by the mitochondrial fission factor dynamin-related protein 1 (DRP1) and mitochondrial fragmentation [59], as well as increased anaerobic energy production and oxidative stress in the heart [63]. The consequences include cardiac ATP depletion and diastolic dysfunction as assessed by echocardiography [63]. Both heart and kidney are characterized by very high mitochondrial densities to satisfy their high energy demand, and thus mitochondrial dysfunction is considered to be a crucial factor in the detrimental progression of the cardio-renal interaction following AKI [63].

The most notable potential biomarkers for AKI-related cardiac injury include the renal protein neutrophil gelatinase-associated lipocalin, which is associated with cardiac fibrosis, fibroblast growth factor 23, which may mediate AKI-induced cardiac damage, and the cardiac injury marker galectin-3 [12]. Other proteins that were recently identified as potential links between heart failure and kidney injury are insulin-like growth factor-binding protein 7 and kidney injury molecule 1 [64]. Finally, AKI-induced IL-6 due to its role in the pathogenesis of atherogenesis [65] may be linked to the increased prevalence of subclinical coronary artery disease (CAD) demonstrated in masters endurance athletes [66].

## Potential Risk Factors Linking Transient Increases of Injury Markers to Permanent Damage

### Aging, Pre-existing Diseases, and Nonsteroidal Anti-Inflammatory Drugs (NSAIDs)

Although the transient increase of cardiac and kidney injury markers in response to EEE is usually reversible and not associated with permanent myocardial and/or kidney damage [45, 46], aging, pre-existing CV, metabolic and/or renal diseases, and drug use/abuse may all contribute to potential long-term adverse clinical consequences [38]. Aging is the most important risk factor for development of CV, metabolic, and other diseases [67, 68]. Even healthy aging is accompanied by a decline in CV, pulmonary, and kidney function, associated with changes in exercise tolerance and recovery characteristics [67, 68].

Although it has been demonstrated that ultramarathon runners (*N* = 1212) on average are healthier when compared with the general population, the prevalence of serious medical issues was shown to be non-trivial [47]. For example, 7.6% of runners reported arrhythmias or irregular heartbeats and another 7.6% suffered from systemic hypertension, but only 3.3% took anti-hypertensive medication [47]. Therefore, based on the well-established blood pressure—heart—kidney interaction, some of those runners may be at an elevated risk of developing CV and/or kidney disease [48]. Furthermore, regarding CV risk factors, for example, exercise-induced hypertension was suggested to increase the prevalence of coronary artery plaque among middle-aged male marathon runners [69]. Higher coronary artery calcium was demonstrated in older male athletes when compared with sedentary males with a similar risk profile [66]. Consequences of recent viral or bacterial infections and/or other pre-existing conditions may promote AKI and associated long-term consequences [38]. Intriguingly, in a recent meta-study of amateur runners (with races ranging from 10 km up to ultramarathons; mean age and range: 41 years, 15.7–63.4 years), both younger age and higher body mass index were correlated with greater cTnI release [70], possibly due to greater cardiac demands secondary to more intense efforts and larger body size. In contrast, reduced myocardial stress could be associated with slower running speed and/or chronic exercise adaptations to exercise among veteran endurance athletes.

The use of NSAIDs is common among marathon and ultramarathon runners, 48.3% of whom report NSAID use for at least one of the time-points considered: before, during, and/or immediately after the competition [71]. Although controversy exists regarding their potentially negative effects in athletes, the chronic use of high-dose NSAIDs is harmful to renal function [42]. Adverse consequences of long-term NSAID use are well established among elderly individuals and those with specific comorbidities [72], which may in some cases also be true for extreme endurance athletes [38].

### Sex

The percentage of female ultra-marathon competitors increased from close to 0 in the 1970s to about 10–20% in 2000 and remained fairly stable subsequently [73, 74]. Since then, physiological differences between the sexes in EEE-relevant aspects are being increasingly acknowledged [75–78]. Female advantages may include greater fatigue resistance and substrate efficiency, as well as lower energetic demands [79]. Conversely, men on average have greater oxygen-carrying capacities and fewer gastrointestinal distress symptoms during ultra-endurance exercise [79]. In addition, thermoregulation following exercise-induced dehydration may differ between sexes, with a potentially higher rate of core temperature increase in females [80].

A recent prospective study (128 runners) revealed sex as one of the major risk factors for meeting diagnostic criteria of AKI during an ultra-marathon [81]. These authors reported that AKI was significantly associated with female sex, lower pack weight, and percentage weight loss (respective odds ratios and 95% confidence intervals (CIs) were: 4.64, 2.07–10.37; 0.71, 0.56–0.91; 0.87, 0.78–0.97) [81]. While male sex generally represents an independent risk factor predisposing to AKI [62], an increased risk of ultra-endurance race-related AKI for women has also been reported [81]. Conversely, men are more at risk for subclinical CAD [66] and permanent cardiac damage due to EEE, as recently reviewed [82]. Generally, the overall incidence of fatal events, such as sudden cardiac deaths, related to exercise is much lower in women compared to men [83]. In fact, the incidence of sudden cardiac arrest during half and full marathons is 0.9 per 100,000 participants for men and 0.16 for women [84].

The mechanistic underpinnings for these sex differences are not well understood but have been hypothesized to be primarily linked to sex hormones [85]. The following putative mechanisms have been recently suggested [86]:

- Potentially protective effects of estrogen in inhibition of myocardial hypertrophy, which conversely is promoted by testosterone.
- Different sex-specific enzyme activities as suggested by animal experimentation, with higher activities of some enzymes involved in energy substrate uptake, such as protein kinase B (AKT) and glycogen synthase kinase-3-beta (GSK 3), that might result in cardio-protection.
- Sex influences in the prevalence of genetic polymorphisms of RAAS, which may also explain the differential vulnerability.

In contrast to male endurance athletes [30], data on myocardial fibrosis in female athletes are scarce. One study assessed 83 asymptomatic triathletes (> 10 training hours per week; 65% males) and detected non-ischemic myocardial fibrosis (by late gadolinium-enhancement) in 17% of males but in none of the 29 female triathletes [87]. These authors suggested that in addition to the potential role of testosterone, differences in blood pressure and race distances (both tended to be lower in female athletes) might contribute to the observed differences between sexes.

### The Role of Event-Related Characteristics

Event-specific risk factors like the duration and intensity of endurance exercise are thought to be important modulators of cardiac injury and biomarker levels [24]. A recent prospective study [88] reported more evidence of myocyte necrosis (cTnI) and cardiac congestion (NT-proBNP) in ultramarathoners compared to marathoners. Similarly, a systematic review revealed ultra-endurance runners to be much more commonly affected by exertional rhabdomyolysis and AKI than athletes competing in other endurance races [89]. In contrast, an older meta-analysis reported an inverse correlation of endurance event distance and troponin levels [19]. These unexpected results may be due to different fitness levels and event characteristics among study participants. Other authors report exercise intensity as the primary modulator of cTnI concentrations [90].

The distance seems to be one key variable especially in mountain ultramarathons [91]. In these events, alternating prolonged uphill and downhill portions are associated with repetitive eccentric contractions that lead to muscle damage, inflammatory responses, and edema in the lower limbs. Interestingly, the inflammatory [e.g., C-reactive protein (CRP)] and muscle damage (e.g., CK) biomarkers were increased less after 330 km [92] than after 170 km [93], probably because the downhill velocity was lower in the former event. Similarly, the acute impact of ultra-marathon running on markers of cardiac fatigue has been shown to be positively influenced by the intensity and as a consequence inversely by the distance; i.e., the longer the ultramarathon the less the cardiac fatigue [91]. Maufrais et al. reported that the transient dysfunction of the LV, generally observed after an intense endurance exercise such as marathon [94], ironman triathlon (with an estimated intensity corresponding to 65–85% of the individual aerobic capacity, VO_2_max) [95] or after an ultramarathon of 170 km [96], was not present after an ultramarathon of 330 km (at about 50% of the velocity related to the individual VO_2_max) [91]. This also may apply to diagnostic criteria for AKI, as a more pronounced temporary decline in renal function was observed after completing a 100 km race compared to a 308 km race [97]. Furthermore, extreme environmental conditions, in particular heat, were shown to aggravate exercise-related adverse renal effects [98]. Findings of combined evaluation of cardio-renal injury markers after (ultra)marathon running are discussed below (Sect. 3.5.1).

### The Role of the Overall Training Load and Participation Frequency in Extreme Endurance Events

Accumulating data suggest a potential role of chronic high-volume/intensity training and participation in extreme endurance events for CV damage in certain athletes [99]. For example, 12% of apparently healthy marathon runners (*N* = 102, 50–72 years of age), who had completed at least five marathons during the past 3 years, presented with patchy myocardial scarring, a rate threefold higher than that in the 102 age-matched control individuals [100]. Such unfavorable consequences include pathologic structural remodeling of the heart and large arteries due to repetitive injuries over years, triggered by the acute volume overload of the atria and right ventricle and transiently impaired RV systolic function after each race or intense training session [8]. Patchy myocardial fibrosis (particularly in the atria, the interventricular septum, and the right ventricle) may result in and constitute the basis for AF/flutter and ventricular arrhythmias [101]. Many years of endurance exercise have indeed been shown to be positively associated with the risk of AF and atrial flutter [102]. In this study the adjusted odds ratios per 10 years of regular endurance exercise were 1.16 (95% CI 1.06–1.29) for AF and 1.42 (1.20–1.69) for atrial flutter [102]. A recent study [103] focused on the potential impact of training characteristics on atrial arrhythmias in long-term endurance athletes. Based on multivariable analysis (adjusted for age), these authors identified accumulated training duration, but not specific training characteristics like frequency or intensity of endurance exercise, as the only significant predictor of AF [103]. AF was present in 14% of athletes reporting 0–10 years of running and in 44% of those reporting more than 30 years of running [103]. On the other hand, insufficient training may also increase the risk for cardiac dysfunction and injury, for example after the completion of a marathon [94]. The increased fatality risk in first-time marathon participants is likely due to underlying myocardial and atherosclerotic CAD in combination with inadequate preparation or poor training [104].

### Risk Factors, Repetition, Regeneration: The 3 Rs in Long-Term Cardiac Damage in Endurance Athletes?

A combination of risk factors with repeated endurance training and competitions (and associated increases of cardiac and renal injury markers) and possibly insufficient regeneration might synergistically favor the development of long-term sequelae (Sects. 3.5.1–3.5.3).

#### Is There an Association Between Cardiac and Renal Injury Markers Following EEE?

A combined evaluation of cardio-renal injury markers was performed in a prospective observational study of participants in the 2007 Perth Marathon (Western Australia) [11]. In this study, troponin elevations were observed in 32% of included marathon finishers. Increased creatinine levels were strong predictors, suggesting a pathophysiological connection between troponin elevations and reduced renal clearance [11]. Among 167 participants of the Berlin marathon (2006 and 2007), more than 30% exhibited a significant increase in cardiac biomarkers (cTnT, NT-proBNP) and a similar proportion showed a marked decrease (> 25%) in cystatin C estimated eGFR after finishing the race, but all parameters returned to baseline 2 weeks post-marathon [54]. Markers for potential cardio-renal injury in adolescent amateur runners seem to be similarly elevated as in adults [105]. Cardio-renal effects due to ultramarathon running (63 km distance and 1800 m altitude difference) were also studied in Mexican Tarahumara, who are accustomed to such ultra-distance races [106]. Markers for kidney (copeptin-ultra sensitive) and cardiac injury (high-sensitivity cTnT) remained elevated 24 h post-race and both markers were inversely related to left-ventricular ejection fraction [106]. While those observations indicate a co-occurrence of and some interrelation between cardiac and kidney injury markers, they do not provide a strong link to permanent damage. However, the existence of such a link cannot be totally dismissed, due to the known adverse cardiac consequences of AKI [12] and the elevated risk of AF or atrial flutter in some life-long endurance athletes [102, 103].

#### Mitochondrial Dysfunction, Renin–Angiotensin–Aldosterone System (RAAS), Oxidative Stress, and Inflammation

Several mechanisms may link EEE, increased kidney and cardiac injury markers, and long-term cardiac damage. For example, mitochondrial dysfunction, including mitochondrial fragmentation, is recognized as a key factor in AKI [107] and in cardio-renal syndromes [62]. On the other hand, mitochondria strongly benefit from endurance exercise. Recent excellent reviews summarize the current body of knowledge on beneficial effects of endurance exercise on mitochondrial functions in skeletal muscle, which include increases in mitochondrial respiration and mitochondrial protein content [108]. In contrast to the mitochondrial fragmentation in cardio-renal syndromes [59], endurance exercise exerts pro-fusion [109] and anti-fragmentation [110] effects on mitochondria. Importantly, not only skeletal muscle mitochondria but also mitochondria in other organs including the heart and kidney are affected by endurance exercise, for example by preventing the decrease in mitochondrial NADH-cytochrome-C reductase and cytochrome oxidase activities in those organs [111]. Although the mechanisms for the transfer of the mitochondrial effects to tissues distant from skeletal muscles are not fully understood yet, the pronounced impact of exercise on mitochondria in many tissues becomes increasingly established and may explain many of the health effects of exercise [112]. There is growing evidence supporting an important role of exercise-induced protective effects (e.g., in the prevention of metabolic, cardiovascular, and neurodegenerative diseases), at least partly mediated by autophagy, mitophagy, and mitochondrial biogenesis [112]. Evidence is also accumulating that chronic moderate endurance exercise can be protective for AKI [113].

Many of the exercise benefits seem to rely on mitochondrial hormesis (i.e., mitohormesis), a phenomenon in which mild mitochondrial stress induces subsequent enhanced mitochondrial resilience [114]. This means, by implication, that a mitochondrial challenge is required to obtain these benefits and that, if the organism at baseline does not have the resilience to deal with such a challenge, mitochondrial damage can ensue [114]. Heart and kidney mitochondrial damage following extreme endurance racing is supported by observations of mitochondrial damage with excessive exercise [115, 116]. A recent study confirmed the well-known involvement of oxidative stress and inflammatory mechanisms [9] in triggering endothelial and renal dysfunction induced by marathon and ultramarathon running [117]. These disturbances rapidly normalized, returning to baseline within 2 days after the race in this study [117]; however, in other studies the stress markers remained high for longer durations post-exercise [44]. Notably, in a mouse model of arrhythmogenic cardiomyopathy, endurance exercise triggered permanent cardiac damage via calcium overload and calpain-1 induced cleavage of mitochondrial-bound apoptosis-inducing factor, leading to cardiomyocyte death [118].

High respiratory activity of mitochondria—such as occurs during endurance exercise—and in particular mitochondrial damage (e.g., due to excessive exercise) are associated with high levels of mitochondrial reactive oxygen species (ROS) [119]. While physiological levels of ROS mediate beneficial adaptations to exercise [120], excessive amounts of ROS cause oxidative damage and are fundamentally involved in cardio-renal syndrome pathogenesis [121]. Oxidative stress can cause renal dysfunction that in turn aggravates mitochondrial dysfunction, thereby creating a vicious cycle that may be involved in kidney diseases [122]. In particular, excessive RAAS-activation is linked to mitochondrial dysfunction and consequential oxidative stress. Chronic RAAS activation can have detrimental consequences on kidney and heart [123]. Activation of RAAS plays a key role in the pathogenesis of the cardio-renal syndrome, for example by causing oxidative stress and uremic toxin overload [123].

Regular PA is associated with reduced RAAS-activation in response to exercise [124], but acute exercise increases angiotensin levels [124]. High levels of angiotensin II, especially in aged individuals, may cause mitochondrial dysfunction, oxidative stress, and inflammation [125], as well as apoptosis in both the kidney [126] and the heart [127]. In line with these findings is the discovery of direct modulation of mitochondria by mitochondrial angiotensin II receptors [128]. Angiotensin II further stimulates aldosterone release, which in turn is linked to an increased release of galectin-3 [121]. A higher response of aldosterone to angiotensin II in female as compared to male rodents [129] may be involved in the reported increased AKI-susceptibility in EEE in women [81].

Mitochondrial dysfunction resulting from excessive exercise thus may be implicated in the etiology of AKI and cardio-renal disease, in which the role of mitochondrial damage is increasingly recognized. The release of damage associated molecular pattern molecules (DAMPs) from stressed mitochondria is further associated with inflammatory responses, which are also commonly observed in extreme endurance athletes (increased CRP, neutrophils and various inflammatory cytokines) [92]. Several changes of blood biomarkers in extreme endurance athletes reflect a hemodilution (i.e., decreased protein concentrations [92] and hematocrit [130]) that likely arises from inflammatory responses and muscle damage induced by repetitive eccentric contractions [131]. It is noteworthy that such hemodilution has been observed predominantly for long durations of > 5 h of running time (thus longer than a usual marathon time).

#### Cumulative Injuries Due to Repeated Endurance Exercise? The Role of Regeneration

Incomplete resolution of renal dysfunction, for example due to repeated AKIs, may be associated with the development of chronic comorbidities like CKD and CV disease (CVD) [132]. Pathophysiological mechanisms involved in the transition from AKI to chronic conditions are thought to include endothelial dysfunction, incomplete regeneration of tubular cells, epigenetic changes due to AKI, persistent chronic inflammation, mitochondrial dysfunction, chronic RAAS activation, as well as cell and tissue senescence [132]. Pre-existing comorbidities like diabetes and/or hypertension but also older age are risk factors for the transition from AKI to CKD and related CVD [132]. CKD is associated with elevated markers of oxidative stress and inflammation [133]. Together with endothelial dysfunction, those risk factors may be key players in the development and progression of atherosclerosis and associated CVD [134, 135].

Furthermore, repeated excessive endurance exercise has been linked to incomplete repair of usually transient exercise-induced CV damage and ultimately the development of patchy cardiac fibrosis in veteran extreme endurance athletes [99]. Insufficient regeneration time between repeated endurance competitions and training is a plausible explanation for incomplete repair and long-term effects of exercise-induced kidney damage and myocardial injury. Finally, reparative processes of heart and kidney may be temporally different. This depends on the genetic background and individual experiences, including exposures to repeated episodes of severe stress, in particular if they differentially affect either the myocardium or kidneys. Such individual factors may explain how repetitive challenges, for example by EEE, could evoke either kidney damage alone, heart damage alone, or both together. Long-term prospective studies investigating the potential of accumulating transient damage of heart and kidneys as causative factors for rare permanent cardiac damage in long distance runners are important to better understand the role of the cardio-renal interplay in pathogenesis.

## Conclusions and Recommendations

Despite the indisputable beneficial consequences of regular exercise, a small number of predominantly male and older long-term endurance athletes have an increased risk of certain CVDs, i.e., myocardial fibrosis, coronary artery calcification, and associated cardiac arrhythmias (e.g., AF, atrial flutter, and ventricular ectopy)], for insufficiently understood reasons [66, 99, 100, 102, 103]. Some of these events are likely related to subtle pre-existing comorbidities, but an unfavorable interplay between repeated (over years)] cardiac and renal injuries related to chronic EEE and incomplete recovery is plausible [38, 100]. Based on the intimate link between heart and kidney diseases [12], it is reasonable to speculate that excessive endurance sport may induce adverse cardio-renal interactions that under specific, hitherto undefined conditions could result in persistent cardiac damage [13]. The increase of cardiac and/or renal injury markers following marathon and ultramarathon events appears to be largely reversible and innocuous, but may result in persistent cardiac damage in some life-long endurance athletes [99, 103].

**Fig. 5:**
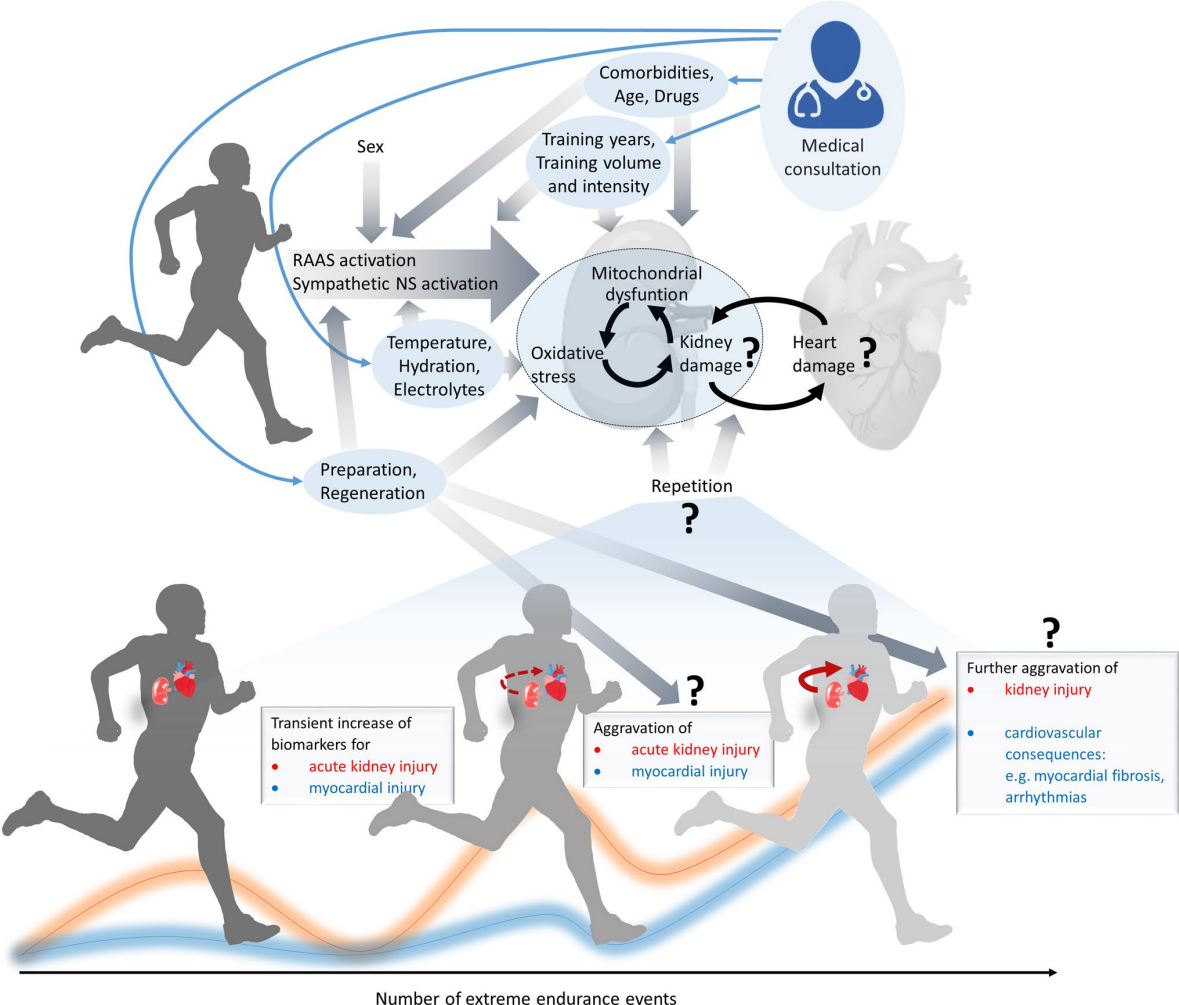
Modulators of potential adverse cardiac and renal effects of extreme endurance exercise (EEE) and potential accumulation of damage. While the mechanistic links require a better understanding, medical consultation may reduce the risk of long-term damage by especially taking into account the highlighted risk factors (blue ellipses). *RAAS* renin–angiotensin–aldosterone system, *NS* nervous system

Such risk factors include pre-existing acute or chronic diseases, repetitive cardio-renal damage, abuse of cardio- and/or nephrotoxic drugs, and specific characteristics of endurance training and competitions (e.g., training years, training volume and intensity, type and frequency of competitions, environmental conditions, and recovery characteristics) [38, 103]. Potential modulators, which may represent important considerations for medical consultation of extreme endurance athletes, are depicted in Fig. 5.

Specifically, increasing discomfort or pain during and/or after endurance events, pre-existing or emerging CV and/or kidney diseases, and regular or acute intake of nephrotoxic drugs such as NSAIDs should be considered when making recommendations about safe doses of exercise for extreme endurance athletes. Celecoxib might be considered a safer NSAID than others for EEE as it was shown to produce fewer renal events than ibuprofen and a favorable trend when compared with naproxen [136]. Adequate preparation not only allows the athlete to assess their own capacities but also conditions the person for subsequent competitions, including the downregulation of excessive RAAS activation in response to exercise [124, 137]. The preparation should be according to the expected duration and intensity of the event, and strategies for hydration and electrolyte repletion should be developed. Given the possibility that repeated EEE might be involved in the pathogenesis of cardiac injury in extreme endurance athletes, a particular emphasis should be placed on allowing sufficient time for rest and recovery between competitions and also between intensive training sessions. Injuries, infections, and increasing age may prolong the required time for complete repair of exercise-induced transient organ damage, for example the skeletal muscles but also kidneys and the CV system.

To what extent long-term pathophysiological mechanisms induced by cardio-renal interactions are really at work in extreme endurance athletes remains speculative and must be evaluated in future studies. For instance, this is the objective of a prospective long-term follow-up analysis, the Pro-MagIC study [138]. Finally, all these efforts will help to provide the foundation for improved counseling, training, and care of current and prospective athletes who are interested in EEE. This will help them reach an informed decision on whether to participate in such sports, and thereby to realize the full benefits of endurance exercise on long-term CV health outcomes.
